# Validation of the Child Oral Health Impact Profile (COHIP) into Sinhala for Adolescents with Cleft Lip and Palate

**DOI:** 10.1177/10556656261441810

**Published:** 2026-04-13

**Authors:** Inoka Mirihagalla Kankanamalage, Wendy Cohen, Joanne Cleland

**Affiliations:** 1 Department of Psychological Sciences and Health, 3527 University of Strathclyde, Glasgow, UK

**Keywords:** cleft lip and palate, quality of life, adolescents, child oral health impact profile, translation and validation

## Abstract

**Objective:**

To translate, culturally adapt, and validate the COHIP for Sinhala-speaking adolescents aged 12 to 15 years with cleft lip and palate (CLP).

**Design:**

This mixed-methods study followed a systematic translation and cross-cultural adaptation process to ensure equivalence between the original instrument and its Sinhala version. The Child Oral Health Impact Profile (COHIP) was translated into Sinhala using a forward-backward-forward procedure conducted by a bilingual expert panel, and linguistic and conceptual equivalence was confirmed prior to piloting.

**Participants:**

Three hundred forty typically developing (TD) adolescents and 45 adolescents with repaired CLP (12-15 years) completed the COHIP-S. Participants were randomly selected from 2 state schools in the western province and speech and language therapy clinics in Sri Lanka.

**Main outcome measure:**

Child Oral Health Impact Profile—Sinhala (COHIP-S)

**Results:**

The translation and adaptation process yielded a culturally appropriate Sinhala instrument. The COHIP-S demonstrated strong psychometric properties, including face, convergent, and discriminant validity, as well as high internal consistency. Cronbach's alpha values were 0.75 for the TD group and 0.76 for the CLP group, indicating that the instrument is suitable for use among both TD adolescents and those with CLP.

**Conclusion:**

The COHIP-S is a reliable and culturally valid tool for assessing oral health–related quality of life among Sinhala-speaking adolescents, including those with CLP.

## Introduction

Cleft lip and palate (CLP) are common congenital craniofacial conditions^1^ requiring multiple surgical and nonsurgical interventions throughout childhood. Individuals with CLP may experience speech and language difficulties, hearing problems, and structural complications that affect feeding and intelligibility.^2,3^ Psychosocial challenges, including reduced self-esteem and social stigma, have also been reported.^4^ Such psychosocial difficulties can limit educational, occupational, and social opportunities. Social relationships, particularly during school years, can be strained by teasing and bullying.^5^ These experiences, compounded by societal stigma and unwanted attention, may contribute to heightened stress, anxiety, and depression.^6^ When speech or hearing problems coexist, psychosocial difficulties are often exacerbated.^7^ Collectively, these challenges underscore how CLP affects health-related quality of life (HRQoL) and oral HRQoL (OHRQoL) across functional, emotional, and social domains. Therefore, assessing quality of life (QoL) in children and adolescents with CLP is an important component of comprehensive cleft care.

Adolescence is a critical period for self-esteem and identity development, and individuals with cleft conditions may still experience challenges with speech, appearance, and social interactions despite successful repair.^8^ Research has shown that some adolescents continue to face stigma, communication difficulties, and reduced social confidence, negatively influencing emotional health and educational outcomes.^9^ Evaluating QoL helps clinicians identify these issues early and provide multidisciplinary support, including speech and language therapy and psychological counseling.^10^ Therefore, QoL assessment ensures a holistic approach to care, addressing not only physical outcomes but also emotional and social adaptation.^11^

QoL in general is a subjective and multidimensional concept that gains meaning primarily at the personal level, as it reflects an individual's perceptions, values, and experiences.^12^ OHRQoL is defined as an individual's subjective evaluation of how their oral health affects their overall well-being, daily functioning, and QoL. It focuses specifically on the functional, psychological, and social impacts of oral health conditions.^13^ OHRQoL is a specific component of HRQoL and contributes significantly to an individual's overall perception of health and well-being, particularly in individuals with conditions affecting oral health such as CLP.^14,15^ The understanding and evaluation of QoL vary across different cultural and social contexts, since what constitutes “a good life” differs according to cultural norms, beliefs, and expectations.^16^ Therefore, when assessing QoL or related constructs such as HRQoL or OHRQoL, any measurement tools need to be adapted and validated to the sociocultural environment in which they are used. This ensures that the instruments are linguistically appropriate, culturally relevant, and psychometrically valid for the target population.^17^

Existing literature reports a wide variation in approaches used to assess QoL among children and adolescents with CLP, including standardized HRQoL and OHRQoL instruments and interviews. While several studies employed generic HRQoL measures such as the Pediatric Quality of Life Inventory (PedsQL),^18,19^ others used oral health–specific QoL instruments like the Child Oral Health Impact Profile (COHIP), Oral Impacts on Daily Performances (OIDP), and Oral Health Impact Profile-14 (OHIP-14).^20–26^ Among these, the COHIP^27^ has emerged as one of the most frequently used and validated condition-specific instruments for the CLP population.

The COHIP, developed by Broder and Wilson-Genderson,^27^ is a validated instrument for measuring OHRQoL in children and adolescents aged 8 to 15 years across various oral health conditions, including CLP. The 34-item questionnaire assesses functional well-being, social-emotional well-being, oral health, school environment, and self-image, incorporating 28 negative and 6 positive constructs.^27^ Compared with generic QoL tools, COHIP more accurately captures the subtle psychosocial and functional impacts associated with CLP.^4,28^ It uniquely includes positive aspects of oral health, such as confidence and attractiveness, and a school environment dimension relevant to school-aged children.^29^ Its robust psychometric properties, cross-cultural adaptability, and sensitivity make it the most widely used and effective self-report tool for evaluating OHRQoL in children and adolescents with repaired CLP.^27,29,30^ The COHIP has been translated and validated in multiple languages, including Dutch,^31^ French,^29^ Korean,^32^ Persian,^33^ and Vietnamese.^34^

The current study is a translation and validation of the COHIP into Sinhala, the main language spoken in Sri Lanka. Sinhala is an Indo-Aryan language, written in the Sinhala script. It has around 16 million speakers, mostly concentrated in Sri Lanka. There are few validated questionnaires or standardized assessments for use in Sinhala-speaking cleft clinics. To our knowledge, PedsQL, a generic HRQoL questionnaire,^35^ and the OIDP, an OHRQoL assessment^36^ were translated and validated into Sinhala with school-aged typically developing (TD) and asthmatic adolescents, but not with adolescents with repaired CLP. Although both generic QoL and HRQoL instruments have been used, there is a notable lack of condition-specific, validated tools designed to capture the unique experiences of children and adolescents with CLP. Generic measures, while useful for comparison across populations, may fail to detect condition-specific impacts.^37^ Similarly, both Eckstein et al^38^ and Klassen et al^4^ emphasized the scarcity of reliable and comprehensive patient-reported outcome measures for CLP. It is therefore clear that an easy-to-use self-reported measure of OHRQoL translated into Sinhala for use with speakers with CLP, and potentially other types of oral health conditions, would be a valuable addition for multidisciplinary cleft care teams and researchers in Sri Lanka.

Therefore, this study aimed to translate the COHIP into Sinhala and investigate the cross-cultural validity of the COHIP-S scale and evaluate its psychometric characteristics among Sinhala-speaking adolescents with repaired CLP living in Sri Lanka.

## Method

### Overview/Design

This study employed a systematic translation and cross-cultural adaptation process to ensure equivalence between the original instrument and its Sinhala version. The COHIP^27^ was translated into Sinhala (COHIP-S) using the standard forward-backward-forward method and following the cross-cultural adaptation guidelines outlined by Guillemin et al.^17^ The translated and culturally adapted COHIP-S was subsequently piloted and validated with adolescents with CLP and TD peers. The PedsQL-Sinhala, a generic QoL questionnaire, was also administered to the same cohort to further examine the construct validity of the COHIP-S. Ethical approval for the study was obtained from University of Strathclyde, Glasgow, UK, and Lady Ridgeway Children's Hospital, Colombo, Sri Lanka, ethics committees.

## Materials

### COHIP

The original English COHIP questionnaire assesses OHRQoL in children and adolescents, including those with CLP. It contains 34 items: 28 on negative impacts and 6 on positive perceptions across 5 subscales (oral health, functional well-being, social-emotional well-being, school environment, and self-image). In addition, there are 2 items regarding treatment expectations and 1 global health perception item.

### PedsQL Inventory

The PedsQL^39^ is a 23-item generic HRQoL measure suitable for healthy children and adolescents, as well as those with acute or chronic health conditions. The instrument includes parent and child forms and assesses 5 domains: physical, emotional, psychosocial, social, and school functioning, and developmentally appropriate versions are available for children aged 2 to 4, 5 to 7, 8 to 12, and 13 to 18 years. The PedsQL has been translated into multiple languages, including Sinhala, for use with adolescents aged 12 to 15 years^40^ in Sri Lanka.

### Procedure for Translating COHIP into Sinhala

Permission to translate the COHIP into Sinhala was obtained from the original author, Dr Hillary Broder. The translation followed a systematic approach based on the guidelines by Guillemin et al^17^ and the World Health Organization's protocol for instrument translation and adaptation.^41^ The COHIP was translated using the forward-backward-forward method, which involved 3 independent translators.

**Step 1—first forward translations:** 2 bilingual, qualified speech and language therapists (SLTs) whose first language was Sinhala independently translated the English version of the COHIP into Sinhala. They considered all aspects of the original instrument, including instructions, design, headers, and footers, while following the guidelines of Guillemin et al^17^ and the WHO translation and cross-cultural adaptation protocol. Linguistic and cultural factors were carefully considered to ensure that the meaning and concepts of the original scale were preserved and that the language would be acceptable to a broad audience.

**Step 2—reconciliation:** The 2 forward translations were reviewed together, and a reconciled forward Sinhala version was produced, meeting the linguistic and conceptual equivalence requirements of the original English draft.

**Step 3—backward translation:** The reconciled Sinhala translation was translated back into English by a third bilingual SLT, whose first language was English and who was blind to the original COHIP version.

**Step 4—final forward translation:** All 3 translators reviewed the forward and backward translations as a committee, identified discrepancies with the original COHIP, and resolved them using structured discussion techniques. The final forward Sinhala version maintained conceptual equivalence while ensuring linguistic appropriateness and comprehensibility for a wide range of speakers. The questionnaire was then formatted according to the COHIP standard format, with guidance from the original author, and received approval confirming completion of the translation process prior to piloting and validation.

### Piloting

The final forward Sinhala translation of the COHIP was piloted with 5 TD adolescents and 2 adolescents with repaired CLP, aged 12 to 15 years. These participants were chosen as a representative match with the COHIP study participant group and the general wider population. The pilot testing assessed equivalence between the source and translated versions, using the probe technique to confirm face validity (by asking verbally) and ensure questions were understandable and acceptable. Minor language and cultural adaptations were made before applying the COHIP-S in the larger study.

SLTs in the cleft clinic were consulted throughout the translation process and provided with the final COHIP-S questionnaire. They were briefed on the evidence supporting the use of COHIP in other languages with children with CLP. The SLTs evaluated the appropriateness of the translated language and considered children's feedback on usability, further confirming the clinical feasibility of the COHIP-S questionnaire.

### Participants

Four hundred parents of TD children received study information and consent forms through their schools. Following parental consent and adolescent assent, 340 TD adolescents (12-15 years) participated; 23 incomplete questionnaires were excluded, leaving 317 for analysis (mean age = 13.22, SD = 0.86). SLTs identified 45 adolescents (12-15 years) with repaired CLP attending clinics during the study period. Parents provided written consent, and adolescents provided assent at the clinic. Two incomplete questionnaires were excluded, resulting in 43 CLP participants (mean age = 14.16, SD = 1.05). The TD group comprised 180 males (53%) and 160 females (47%), while the CLP group comprised 18 males (40%) and 27 females (60%).

### Inclusion and Exclusion Criteria

Children whose first language was Sinhala were included. For the TD group, those with chronic medical conditions requiring daily medication, learning or cognitive difficulties, or hearing impairments were excluded after consulting teachers and parents with the assumption that TD children would be scoring 100% in the QoL scale by this age. For the CLP group, participants who had undergone surgical interventions within accepted time frames (primary palatal repair done before age of 18 months prior to first speech assessment) were included, while those with significantly delayed primary palatal repair, associated syndromes, or severe learning disabilities that precluded questionnaire completion were excluded after referring to SLT notes.

### Data Collection

Participants were recruited from urban and nonurban mainstream schools in the western province and from the Speech and Language Therapy Department (Cleft Clinic) at the Lady Ridgeway Children's Hospital, Colombo, Sri Lanka, one of the country's leading centers for pediatric craniofacial care. Participants were chosen randomly from schools and clinics according to the inclusion and exclusion criteria to reduce sample bias. TD participants were randomly selected from each grade, with classes chosen proportionally when grades contained more than 5 classes. Adolescents who received parental consent within the response time of 7 days from the original invitation (85% response rate) and subsequently provided their assent completed the COHIP-S and the PedsQL-Sinhala questionnaires during class time. They were instructed to respond independently to minimize response bias, and the investigator remained present to provide clarification when required. Completion took approximately 30 min, with an additional 5 to 10 min allowed when necessary.

SLTs identified 45 adolescents aged 12 to 15 years with repaired CLP who were receiving follow-up care during the study period. The SLTs reviewed participant suitability with the investigator. Children with CLP, and their parents, were invited to participate while attending hospital clinics. At the clinic, parents received study information and provided written consent for their child to participate for the study, after which their children were asked to provide assent.

While waiting for their SLT or orthodontic appointments, CLP participants completed paper versions of the COHIP-S and PedsQL-Sinhala questionnaires. The clinic SLT remained available throughout data collection to clarify questions and provide assistance when needed. Participants were asked to complete the questionnaires independently within approximately 30 min, with additional time allowed if required. Data collection for the CLP group was completed within 3 months. All 45 children with repaired CLP invited completed the questionnaires, yielding a 100% response rate.

### Sample Size Justification

Based on Charter,^42^ a sample size of >300 was considered adequate for validity and reliability testing. Prior COHIP studies^29,30,43^ involving 100 to 1000 participants confirmed its validity for 8- to 16-year-olds. In Sri Lanka, the Sinhala PedsQL showed good psychometric properties in 257 adolescents.^35^ As CLP prevalence is low (0.83/1000)^44^ and specialist services are limited, a pragmatic sample of >300 TD adolescents and 45 adolescents with CLP was targeted for this study. At Lady Ridgeway Children's Hospital, Colombo, Sri Lanka, children with repaired CLP receive follow-up speech therapy until 18 years of age as part of cleft multidisciplinary team (MDT) care or discharge from the MDT. Therefore, this group reflects those with ongoing MDT follow-up rather than the entire cleft population.

### Validation

The assessment of the accuracy and consistency of research instruments, particularly questionnaires following translation and cross-cultural adaptation, is a crucial step in ensuring measurement quality.^17,45,46^ In accordance with these established guidelines, the present study evaluated the reliability (internal consistency) and validity (face, discriminant, and construct validity) of the COHIP-S questionnaire. The COHIP-S questionnaire was validated with TD adolescents and adolescents with repaired CLP aged 12 to 15 years. A consideration was given to previous validity evidence of the COHIP along with piloting the COHIP-S before use in the study to account for any practical issues children reported while completing the questionnaire.

### Analysis

Each questionnaire was scored according to the guidelines for that questionnaire. Children's responses to the COHIP-S and PedsQL-Sinhala were entered into Microsoft Excel™ and subsequently converted into SPSS version 25. Participant responses were analyzed at item and subscale levels. In COHIP, the first 4 subscales are negatively worded (scored from 0 (“never”) to 4 (“almost all the time”)) and reverse-scored so that higher scores indicate better OHRQoL, while the fifth subscale, self-image, items are positively scored (from 0 (“strongly disagree”) to 4 (“strongly agree”)). Total scores could range from 0 to 136 for the overall scale, with higher values reflecting better OHRQoL. Apart from the 5 subscale items, 2 treatment expectation items and 1 global health perception item were analyzed separately. Missing data exceeding 25% per subscale were excluded from the analysis.

Feasibility was assessed via missing values and completion rates, and range via ceiling and floor effects. Non-normal data (Kolmogorov-Smirnov with Lilliefors) were analyzed using nonparametric tests. Item-level correlations and Cronbach's alpha evaluated internal consistency, with *α* > 0.7 considered acceptable. Convergent validity was examined using Spearman correlations among subscales, total scores, and global health, expecting higher correlations between conceptually related scales. Discriminant validity was assessed by comparing COHIP-S scores between CLP and TD groups across ages using the Kruskal-Wallis test, anticipating higher scores in the TD group.

## Results

### Translation and Cultural Adaptations

The Sinhala-English bilingual SLTs reported that it was culturally appropriate to retain the original structure of the questionnaire. In the final forward translation of the COHIP-S, no major modifications were made to the questionnaire's content other than minor wording adjustments to enhance cultural relevance and comprehensibility. Specifically, the Sinhala dictionary translation for the word mouth, /mukəjə/, was replaced with the more commonly used synonym /kɑʈə/, which is familiar in everyday speech and more accessible to children. Similarly, several dictionary-derived terms were substituted with simpler alternatives, and sentence structures were revised to reflect Sinhala grammatical conventions, wherein verbs typically follow nouns or adjectives. After piloting, no further changes were made to the questionnaire, and the translating committee agreed that the COHIP-S achieved cultural appropriateness while maintaining the purpose of the tool.

### COHIP-S Ratings for TD Children

TD participants were distributed across 4 age groups (12-15 years), with group sizes ranging from 51 to 110. Unequal sizes reflected exclusions and variable parental consent. Item-level analysis (Table 1) showed that most TD participants reported no oral health concerns, although some noted problems such as tooth pain, spacing, or bad breath. Eighteen of the 34 COHIP-S items were endorsed by 25% or fewer participants. For the global health item (Q37), none rated their overall health as “poor,” and most selected “good” (39.7%) or “excellent” (38.2%). Responses demonstrated full use of the rating scale, with distributions skewed toward higher OHRQoL. The mean total COHIP-S score for TD adolescents was 118.68 (maximum = 136). Subscale means indicated positive OHRQoL: oral health = 33.54/40, functional well-being = 22.65/24, social-emotional well-being = 29.57/32, school environment = 15.42/16, and self-image = 17.49/24. The school environment subscale was closest to its maximum, while oral health and self-image showed wider score gaps, suggesting the presence of some oral health concerns even among TD adolescents.

**Table 1.** Item-Level Analysis: Frequency Distribution (*n* (%)) of COHIP-S Responses in TD and CLP Adolescents.

Question	TD (*N* = 317)					CLP (*N* = 43)				
	Never	Almost never	Sometimes	Fairly often	All the time	Never	Almost never	Sometimes	Fairly often	All the time
Oral health										
Q1. Had pain in your teeth/toothache	167	91	55	3	1	13	21	8	1	0
	52.7%	28.7%	17.4%	0.9%	0.3%	30.2%	48.8%	18.6%	2.3%	0.0%
Q2. Been breathing through your mouth or snoring	213	49	45	7	3	20	12	6	4	1
	67.2%	15.5%	14.2%	2.2%	0.9%	46.5%	27.9%	14.0%	9.3%	2.3%
Q3. Had discolored teeth or spots on your teeth	217	53	31	10	6	16	16	7	4	0
	68.5%	16.7%	9.8%	3.2%	1.9%	37.2%	37.2%	16.3%	9.3%	0.0%
Q4. Had crooked teeth or spaces between your teeth	217	53	22	9	16	21	10	6	3	3
	68.5%	16.7%	6.9%	2.8%	5.0%	48.8%	23.3%	14.0%	7.0%	7.0%
Q5. Had sores or sore spots in or around your mouth	206	65	38	7	1	22	12	7	2	0
	65%	20.5%	12%	2.2%	0.3%	51.2%	27.9%	16.3%	4.7%	0.0%
Q6. Had bad breath	237	42	29	5	4	25	10	6	2	0
	74.8%	13.2%	9.1%	1.6	1.3%	74.8%	23.3%	14.0%	4.7%	0.0%
Q7. Had bleeding gums	246	35	25	8	3	29	8	6	0	0
	77.6%	11%	7.9%	2.5%	0.9%	67.4%	18.6%	14.0%	0.0%	0.0%
Q8. Had food sticking in or between your teeth	116	85	90	15	11	8	17	14	4	0
	36.6%	26.8%	28.4%	4.7%	3.5%	18.6%	39.5%	32.6%	9.3%	0.0%
Q9. Had pain or sensitivity in your teeth with hot or cold things	167	71	54	11	14	20	17	5	1	0
	52.7%	22.4%	17%	3.5%	4.4%	46.5%	39.5%	11.6%	2.3%	0.0%
Q10. Had dry mouth or lips	156	99	40	13	9	19	20	1	1	0
	49.2%	31.2%	12.6%	4.1%	2.8%	44.2%	46.5%	2.3%	2.3%	0.0%
Functional well-being (because of your teeth, mouth, or face)										
Q11. Had trouble biting off or chewing foods such as apple, carrot or firm meet	270	25	16	3	3	27	9	7	0	0
	85.2%	7.9%	5.0%	0.9%	0.9%	62.8%	20.9%	16.3%	0.0%	0.0%
Q15. Had difficulty eating foods you would like to eat	265	31	21	0	0	18	13	9	3	0
	83.6%	9.8%	6.6%	0%	0%	41.9%	30.2%	20.9%	7.0%	0.0%
Q20. Had trouble sleeping	286	22	7	2	0	38	3	1	1	0
	90.2%	6.9%	2.2%	0.6%	0%	88.4%	7.0%	2.3%	2.3%	0.0%
Q23. Had difficulty saying certain words	260	37	16	3	1	13	19	5	2	4
	82%	11.7%	5%	0.9%	0.3%	30.2%	44.2%	11.6%	4.7%	9.3%
Q25. Have people had difficulty understanding what you were saying	272	31	11	3	0	21	9	6	4	3
	85.8%	9.8%	3.5%	0.9%	0%	48.8%	20.9%	14.0%	9.3%	7.0%
Q27. Had difficulty keeping your teeth clean	266	27	12	7	5	25	6	8	2	2
	83.9%	8.5%	3.8%	2.2%	1.6%	58.1%	14.0%	18.6%	4.7%	4.7%
Social-emotional well-being (because of your teeth, mouth, or face)										
Q12. Been unhappy or sad	242	37	24	6	8	25	7	6	5	0
	76.3%	11.7%	7.6%	1.9%	2.5%	58.1%	16.3%	14.0%	11.6%	0.0%
Q16. Felt worried or anxious	233	48	26	3	7	22	9	6	3	3
	73.5%	15.1%	8.2%	0.9	2.2%	51.2%	20.9%	14.0%	7.0%	7.0%
Q17. Felt shy or withdrawn	269	29	13	4	2	26	9	5	3	0
	84.9%	9.1%	4.1%	1.3%	0.6%	60.5%	20.9%	11.6%	7.0%	0.0%
Q19. Avoided smiling or laughing with other children	278	26	8	4	1	28	7	5	1	2
	87.7%	8.2%	2.5%	1.3%	0.3%	65.1%	16.3%	11.6%	2.3%	4.7%
Q22. Been teased, bullied, or called names by other children	276	26	10	4	1	26	9	3	3	2
	87.1%	8.2%	3.2%	1.3%	0.3%	60.5%	20.9%	7.0%	7.0%	4.7%
Q24. Felt that you look different	239	43	22	8	5	12	13	8	5	5
	75.4%	13.6%	6.9%	2.5%	1.6%	27.9%	30.2%	18.6%	11.6%	11.6%
Q28. Been worried about what other people think	273	28	11	3	2	24	9	8	2	0
	86.1%	8.8%	3.5%	0.9%	0.6%	55.8%	20.9%	18.6%	4.7%	0.0%
Q29. Been upset or uncomfortable with being asked questions	260	28	18	5	6	17	10	9	5	2
	82.0%	8.8%	5.7%	1.6%	1.9%	39.5%	23.3%	20.9%	11.6%	4.7%
School environment (because of your teeth, mouth, or face)										
Q13. Missed school for any reason	288	18	9	2	0	34	5	1	2	1
	90.9%	5.7%	2.8%	0.6%	0%	79.1%	11.6%	2.3%	4.7%	2.3%
Q18. Had difficulty paying attention in school	280	35	1	1	0	30	9	2	2	0
	88.3%	11%	0.3%	0.3%	0%	69.8%	20.9%	4.7%	4.7%	0.0%
Q21. Did not want to speak/read out loud in class	279	28	6	2	2	24	4	7	3	5
	88%	8.8%	1.9%	0.6%	0.6%	55.8%	9.3%	16.3%	7.0%	11.6%
Q30. Did not want to go to school	288	16	10	2	1	38	3	2	0	0
	90.9%	5.0%	3.2%	0.6%	0.3%	88.4%	7.0%	4.7%	0.0%	0.0%
Self-image										
Q14. Been confident	38	15	35	71	158	7	2	2	12	20
	12%	4.7%	11%	22.4%	49.8%	16.3%	4.7%	4.7%	27.9%	46.5%
Q26. Felt that you were attractive	68	49	69	62	69	16	12	9	6	0
	21.5%	15.5%	21.8%	19.6%	21.8%	37.2%	27.9%	20.9%	14.0%	0.0%
	Strongly disagree	Somewhat disagree	Do not agree or disagree	Somewhat agree	Strongly agree	Strongly disagree	Somewhat disagree	Do not agree or disagree	Somewhat agree	Strongly agree
Q31. I have good teeth.	25	14	27	49	202	9	5	8	7	14
	7.9%	4.4%	8.5%	15.5%	63.7%	20.9%	11.6%	18.6%	16.3%	32.6%
Q32. When I am older, I believe that I will have good teeth.	40	21	30	61	165	5	1	8	10	19
	12.6%	6.6%	9.5%	19.2%	52.1%	11.6%	2.3%	18.6%	23.3%	44.2%
Q33. When I am older, I believe that I will have good health.	31	21	24	64	177	5	1	5	10	22
	9.8%	6.6%	7.6%	20.2%	55.8%	11.6%	2.3%	11.6%	23.3%	51.2%
Q34. I feel good about myself.	5	26	26	60	200	4	2	4	9	24
	1.6%	8.2%	8.2%	18.9%	63.1%	9.3%	4.7%	9.3%	20.9%	55.8%
Treatment expectations										
Q35. I will feel better about myself when treatment for my teeth, mouth, or face is completed.	41	16	47	48	165	4	1	2	9	27
	12.9%	5.0%	14.8%	15.1%	52.1%	9.3%	2.3%	4.7%	20.9%	62.8%
Q36. I am nervous (anxious) about the treatment that I need for my teeth, mouth, or face.	193	53	35	19	17	24	4	9	2	4
	60.9%	16.7%	11%	6%	5.4%	55.8%	9.3%	20.9%	4.7%	9.3%
Global health										
	Poor	Fair	Average	Good	Excellent	Poor	Fair	Average	Good	Excellent
Q37. Overall, I feel my health is	0	1	69	126	121	0	0	7	25	11
	0%	0.3%	21.8%	39.7%	38.2%	0%	0.0%	16.3%	58.1%	25.6%

### COHIP-S Ratings for CLP Children

More than half of the CLP participants reported never or almost never experiencing many oral health-related issues (Table 1). However, only 48.9% agreed or somewhat agreed that they had “good teeth” (Q31), and nearly all reported experiencing at least 1 oral health problem at some point. The mean total COHIP-S score for the CLP group was 104.46 (maximum = 136). All subscale scores were lower than those of the TD group: oral health = 31.46/40, functional well-being = 19.27/24, social-emotional well-being = 24.79/32, school environment = 13.74/16, and self-image = 15.18/24. These findings indicate greater impacts across multiple domains over the 3 months prior to assessment. School environment scores were again closest to the maximum, whereas oral health, social-emotional well-being, and self-image showed the largest deficits relative to the TD sample. Responses to treatment-related items were generally positive. For Q35, 62.8% strongly agreed that they felt better after treatment (M = 3.26, SD = 1.25). For Q36, 55.8% reported never feeling anxious about treatment (M = 2.98, SD = 1.35).

### Internal Consistency and Correlation Between Items of COHIP-S

#### Item Correlation (TD)

The mean COHIP-S scores (34 items) were calculated for each participant. The Kolmogorov-Smirnov test with Lilliefors correction indicated non-normal data distribution; therefore, nonparametric analyses were used. Reliability within each subscale was assessed using corrected item total correlations, which were all positive and ranged from *r* = 0.19 to 0.51 (oral health), *r* = 0.22 to 0.43 (functional well-being), *r* = 0.45 to 0.73 (social-emotional well-being), *r* = 0.44 to 0.55 (school environment), and *r* = 0.36 to 0.69 (self-image), demonstrating acceptable internal consistency. Spearman's rank-order correlations between subscale totals and the overall COHIP-S score were moderate to high (*r* = 0.5-0.7, *P* < 0.05), with the strongest relationships observed for the oral health (*r* = 0.774) and social-emotional well-being (*r* = 0.797) subscales (Table 2).

**Table 2.** Spearman's *r* Correlations Between COHIP-S Total and Subscale Scores in TD and CLP Groups.

	Oral health		Functional well-being		Social-emotional well-being		School environment		Self-Image		COHIP-S total score	
	TD	CLP	TD	CLP	TD	CLP	TD	CLP	TD	CLP	TD	CLP
Oral health			.507	.693	.555	.388	.475	.327	.119	.219	.774	.603
Functional well-being	.507	.693			.611	.682	.577	.702	.061	.040	.658	.834
Social-emotional well-being	.555	.388	.611	.682			.635	.740	.177	.096	.797	.851
School environment	.475	.327	.577	.702	.635	.740			.140	.234	.663	.830
Self-image	.119	.219	.061	.040	.177	.096	.140	.234			.567	.359
COHIP total score	.774	.603	.658	.834	.797	.851	.663	.830	.567	.359		

#### Item Correlation (CLP)

Reliability was assessed between items within each subscale of the questionnaire and between the total COHIP score and the subscale scores. Reliability within each subscale was assessed using corrected item total correlations, which were all positive and ranged from *r* = 0.23 to 0.48 (oral health), *r* = 0.15 to 0.63 (functional well-being), *r* = 0.46 to 0.76 (social-emotional well-being), *r* = 0.43 to 0.67 (school environment), and *r* = 0.32 to 0.86 (self-image) within subscale items, demonstrating acceptable internal consistency. Spearman's rank-order correlations between subscale totals and the overall COHIP-S score were moderate to high (*r* = 0.35 to 0.85, *P* < 0.05), with the strongest relationships observed for the functional well-being (*r* = 0.834), social-emotional well-being (*r* = 0.851), and school environment (*r* = 0.830) subscales (Table 2).

#### Internal Consistency (TD)

Results indicated a high level of internal consistency for the overall COHIP-S questionnaire. Cronbach's alpha for the total COHIP-S in the TD sample was 0.75, demonstrating good reliability. When examined by subscale, alpha coefficients were satisfactory for school environment and oral health (>0.70) and excellent for social-emotional well-being and self-image (>0.80). The functional well-being subscale showed a lower yet acceptable value (*α* = 0.60), which remains adequate given the small number of items.^47^ As all subscales recorded alpha values between 0.6 and 1.0, no item removal was required to improve reliability (Table 3). Overall, the COHIP-S demonstrated good internal consistency, with all subscales showing acceptable reliability and only a marginally lower coefficient for functional well-being.

**Table 3.** Cronbach's Alpha Values for the TD and CLP Groups.

Scale	Cronbach's alpha (TD)	Cronbach's alpha (CLP)
Oral health	.75	.72
Functional well-being	.61	.69
Social-emotional well-being	.86	.87
School environment	.70	.71
Self-image	.80	.81
Overall scale	.75	.76

#### Internal Consistency (CLP)

The COHIP-S demonstrated good internal consistency across and within subscales, with stable Cronbach's alpha values when items were deleted. All corrected item total correlations were positive, and no items required removal. Subscale alphas were good for school environment and oral health (>0.70), excellent for social-emotional well-being and self-image (>0.80), and acceptable for functional well-being (α = 0.69). Overall reliability was high for both groups (CLP *α* = 0.76), indicating that the COHIP-S is a reliable tool for assessing OHRQoL among Sri Lankan adolescents with repaired CLP (Table 3).

### Validity of the COHIP-S

The COHIP-S demonstrated strong face validity. An expert committee of bilingual SLTs, a teacher, and a medical consultant confirmed the conceptual and linguistic appropriateness of all items, with no major revisions required after piloting. All participants completed the questionnaire easily, providing clear and relevant feedback, with a 100% response rate and 93.63% valid responses.

#### Convergent Validity

Convergent validity of the COHIP-S was assessed by correlating the total score of 5 subscales (questions 1-34) with self-perceived overall health (Q37) and with the PedsQL-Sinhala in both TD and CLP groups.

In the TD group, both variables were non-normally distributed, Spearman's rank-order correlations were used. The COHIP-S total scores were significantly positively correlated with self-perceived overall health (*r* = 0.33, *P* < 0.001), with the strongest associations observed for functional and social-emotional well-being subscales (Table 4) supporting good convergent validity.

**Table 4.** Correlation Between COHIP-S Subscales and Global Health Perception (TD/CLP).

Global Health Perception Score				
	(TD)		(CLP)	
	*r*s (*P*)	*p*-value	*r*s (*P*)	*p*-value
Total COHIP-S	0.330	0.001	0.341	0.02
Oral health	0.293	0.001	0.377	0.01
Functional well-being	0.312	0.001	0.437	0.00
Social-emotional well-being	0.311	0.001	0.354	0.02
School environment	0.259	0.001	0.218	0.16
Self-image	0.189	0.001	−0.005	0.97

In the CLP group, the total COHIP-S scores were approximately normally distributed, while global health scores were positively skewed (*P* < 0.001); therefore, Spearman's rank-order correlations were used. COHIP-S total scores were significantly positively correlated with global health ratings (*r* = 0.34, *P* < 0.05), with strongest associations observed for oral health, functional well-being, and social-emotional well-being subscales, whereas school environment and self-image correlations were weaker or nonsignificant.

Higher COHIP-S and subscale scores, indicating better OHRQoL, corresponded with higher global health perception ratings, supporting excellent convergent validity.

#### Discriminant Validity

The total COHIP-S score was compared across age groups (12-15 years) in TD adolescents using the nonparametric Kruskal-Wallis test due to non-normal distribution. Scores differed significantly between ages (*H*(4) = 7.788, *P* = 0.05) with mean ranks increasing from 145.01 at age 12 to 179.82 at age 15, suggesting that OHRQoL improves with age.

Contrastingly, COHIP-S total scores among CLP participants did not differ significantly across age groups (12-15 years) based on the Kruskal-Wallis test (*H*(4) = 2.230, *P* = 0.526). The mean ranks were 24.21 for 12-year-olds, 16.69 for 13-year-olds, 25.38 for 14-year-olds, and 22 for 15-year-olds. This result suggests that OHRQoL did not improve or decline with age in adolescents with CLP.

These findings indicate that the COHIP-S demonstrates good discriminant validity, effectively distinguishing participants by age and biological sex. To further assess discriminant validity, COHIP-S subscale and total scores of CLP participants were compared with TD participants. In the TD group, males had higher overall COHIP-S scores (M = 121.68, SD = 12.14) than females (M = 119.33, SD = 13.60), whereas in the CLP group, females scored higher (M = 104.92, SD = 17.24) than males (M = 103.68, SD = 15.97).

Moreover, the mean average scores of the total COHIP and subscale mean scores were statistically compared. As COHIP-S total and subscale scores were non-normally distributed (Kolmogorov-Smirnov, *P* < 0.05), Mann-Whitney tests were used to compare TD and CLP groups. TD participants had significantly higher overall COHIP-S scores than CLP participants (*U* = 3240.5, *P* < 0.001). Similarly, all 5 subscales’ (oral health, functional well-being, social-emotional well-being, school environment, and self-image) scores were significantly higher in the TD group compared to the CLP group (*P* < 0.05 for all; Figure 1), supporting the discriminant validity of the questionnaire.

**Figure 1.**
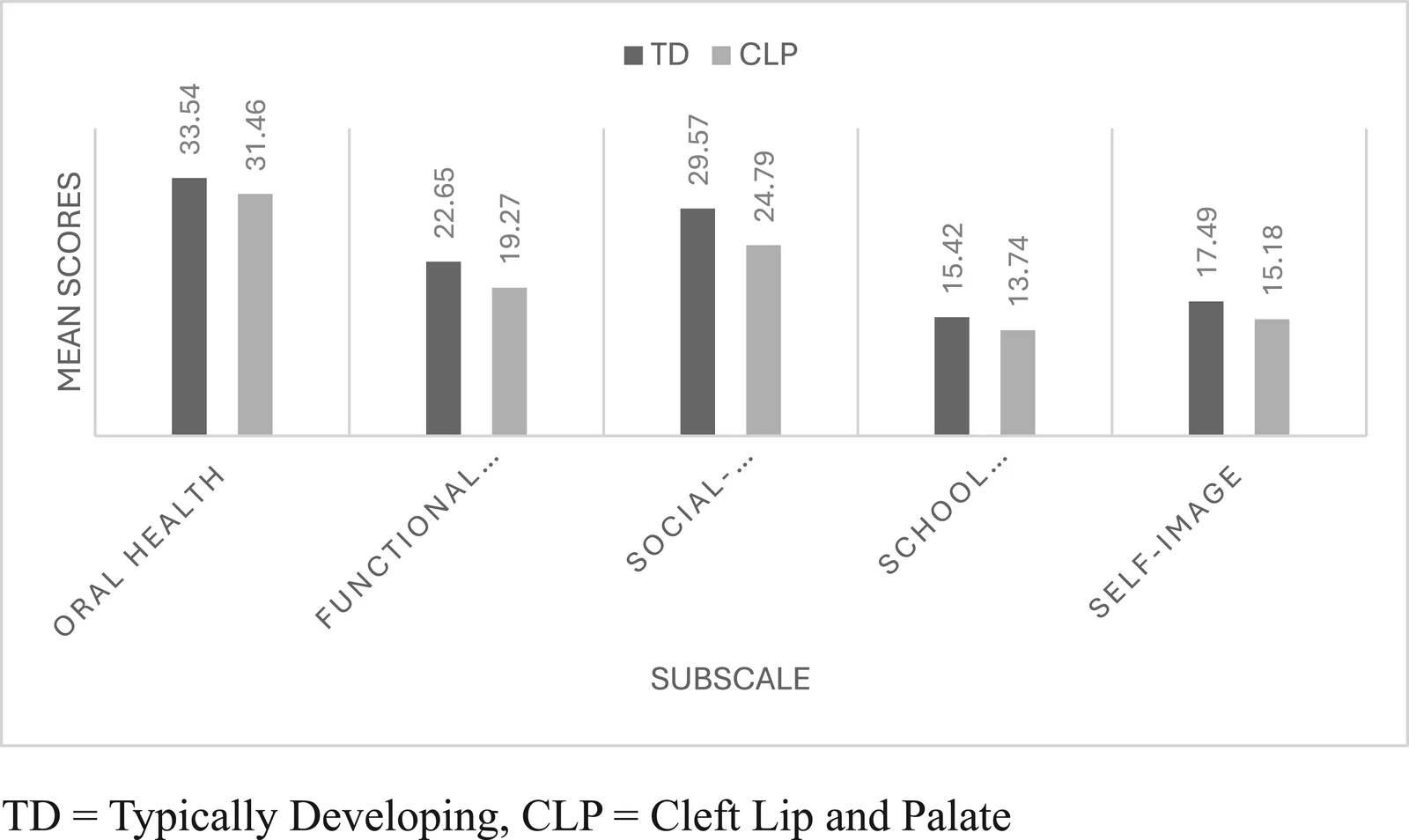
COHIP-S total and subscale scores comparisons between TD and CLP participants. TD = typically developing, CLP = cleft lip and palate.

The final COHIP-S question assesses global health perception but is not included in the total score; it allows participants to rate their overall health. Most TD (40.1%) and CLP (58.1%) adolescents rated their health as good, with over a quarter in both groups rating it as excellent. Mean global health scores were similar between groups (TD = 3.16, SD = 0.77; CLP = 3.09, SD = 0.65), indicating comparable self-perceived overall health despite differences in COHIP-S total scores.

#### Concurrent Validity

The PedsQL-Sinhala questionnaire, validated for 11- to 15-year-old adolescents in Sri Lanka, was used in this study and responses were compared with COHIP-S to assess the concurrent validity of the COHIP-S. Total COHIP-S scores were normally distributed (*P* = 0.20), while PedsQL-Sinhala scores were not (*P* = 0.05); thus, Spearman's correlation was used. A statistically significant positive correlation was observed between the total scores of COHIP-S and PedsQL-Sinhala (*r* = 0.361, *P* < 0.05). Similar significant correlations were found between corresponding subscales of the 2 instruments: functional well-being in COHIP-S with physical functioning in PedsQL-Sinhala; social-emotional well-being in COHIP-S with emotional and social functioning in PedsQL-Sinhala; and the school functioning subscales in both COHIP-S and PedsQL-Sinhala (*P* < 0.05). These results support the concurrent validity of COHIP-S. Both measures captured health-related and OHRQoL issues, but COHIP-S provides a more objective assessment specific to CLP. This was supported statistically by effect size. The comparison for the TD and CLP was larger for COHIP-S (Cohen's d = 0.96) than for the PedsQL-Sinhala (Cohen's d = 0.78), indicating that OHRQoL is more markedly affected (nearly 1 SD difference) in children with CLP than generic HRQoL. The concurrent validity shows the extent of the agreement between 2 measures or assessments taken at the same time. It compares a new assessment with one that has already been tested and proven to be valid. Hence, it can be concluded that COHIP-S is more objective in measuring QoL in CLP population than the established PedsQL-Sinhala measure. These results highlight that while both generic and condition-specific (oral health-related) measures can identify QoL impacts, COHIP-S may be more sensitive for this population.

## Discussion

This study aimed to translate the COHIP English language questionnaire into Sinhala and to validate the questionnaire on both TD adolescents and adolescents with CLP living in Sri Lanka. With only minimal adaptations, COHIP-S achieved cultural appropriateness while maintaining the purpose of the tool.

### Pilot Data of COHIP-S

Nearly all TD adolescents (97.9%) reported at least 1 oral health impact in the past 3 months, indicating that minor oral health issues are common even in normative groups. This aligns with Ahn et al. (2012) but contrasts with the lower prevalence (48%) reported in the Sinhala OIDP validation,^36^ likely due to the broader COHIP-S scale and younger participant age. Despite occasional issues such as tooth pain and dry mouth, most adolescents rated their health as good or excellent, reflecting high OHRQoL. The mean COHIP-S score (M = 118.68) and subscale means were higher than those reported in U.S., Korean, and French studies,^27,29,32^ suggesting that older adolescents exhibit better OHRQoL as permanent dentition stabilizes.^48^ Overall, the findings highlight generally positive oral health perceptions among TD adolescents.

Moreover, all 43 adolescents with CLP in this study (100%) experienced at least 1 COHIP-S impact within the past 3 months, confirming that this group faces more frequent and multifaceted challenges than their TD peers. The most common difficulties reported in this study cohort were speech intelligibility, feeling different in appearance, teasing or bullying, and social anxiety, reflect the strong socio-emotional component of living with CLP which are also reported by Hunt et al.^49^ Oral and functional problems, such as pain, difficulty chewing, and oral hygiene issues, were also reported, consistent with findings by Ward et al.^50^ and Christophe et al.^51^ The mean COHIP-S score for the CLP group (M = 104.46) was notably lower than that of the TD cohort, supporting the hypothesis that adolescents with CLP experience lower OHRQoL. However, slightly higher mean values than those reported in previous CLP studies (eg, Broder & Wilson-Genderson,^27^; Ward et al.^50^) may reflect this cohort's older age, long-term multidisciplinary follow-up, and consistent access to specialized care^20,52^ as this study cohort was chosen from one of the country's leading cleft centers.

### Psychometric Properties of the COHIP-S

The study's results indicate that the COHIP-S is an appropriate and valid instrument for assessing OHRQoL among Sinhala-speaking adolescents in Sri Lanka. It demonstrated excellent reliability and validity in both TD adolescents and those with repaired CLP.

The tool's internal consistency was confirmed with Cronbach's alpha values exceeding 0.7 across all 5 subscales indicating strong item agreement and scale stability. The Cronbach's alpha value was reported as 0.75 for TD adolescents and 0.76 for those with CLP, both exceeding the 0.7 benchmark for good reliability (Field, 2018). Among subscales, functional well-being produced slightly lower alpha values (TD = 0.61; CLP = 0.69), while social-emotional well-being (TD = 0.86; CLP = 0.87) and self-image (TD = 0.80; CLP = 0.81) showed excellent internal consistency.

Moreover, the discriminant validity was established through significantly higher COHIP-S scores among TD adolescents compared to those with CLP (*P* < .01), confirming the instrument's sensitivity to clinical differences. Subgroup analyses revealed minor sex and age variations, reflecting developmental and cultural influences on self-perception and oral health concerns.

In addition, the convergent validity was supported by positive correlations between total COHIP-S scores and self-perceived health ratings in both groups, indicating that higher perceived health was associated with better OHRQoL while face validity was confirmed through participant feedback.

These results align closely with previous COHIP validation studies in English,^27^ Korean,^32^ French,^29^ Amharic,^20^ Chinese,^52^ and Arabic,^21^ all of which demonstrated high internal consistency (Cronbach's α > 0.75) and strong validity evidence.

Overall, the findings demonstrate that the COHIP-S is a reliable, valid, and culturally appropriate measure for evaluating OHRQoL among Sinhala-speaking adolescents. Its comparable reliability to international versions further supports its use in both clinical practice and research within Sri Lanka.

### Limitations and Future Research

This study was part of a larger study investigating QoL of adolescents with repaired CLP in Sri Lanka; hence, the study faced some unavoidable limitations. Data collection in Sri Lanka was restricted to a short time period, limiting the number of age-appropriate CLP participants and contributing to uneven age group distribution. The lack of recent prevalence data for CLP in Sri Lanka made it difficult to determine a fully representative sample. CLP participants were recruited from a leading hospital in the capital that provides specialized services not widely available in other healthcare settings; therefore, the findings cannot be generalized to all adolescents with repaired CLP in the country, particularly those receiving care in nonspecialized or resource-limited facilities. Additionally, participants were recruited during routine SLT and orthodontist clinic follow-up visits, which may have introduced selection bias by overrepresenting adolescents receiving cleft MDT care. Hence, future multicenter studies involving diverse healthcare settings and including adolescents not currently receiving MDT care would provide a more comprehensive understanding of QoL outcomes in this population.

This study lays the groundwork for future research. Similar methodologies can be applied to adapt and validate assessments to enhance clinical resources. Research is needed to translate and validate these tools into Tamil (Sri Lanka's second language) and to evaluate the COHIP-S further, including test-retest reliability and application across a broader age range. Additionally, adapting and validating the COHIP short form could provide a more time-efficient tool for both clinical and research purposes, further supporting quality care for children with CLP in Sri Lanka.

## Conclusion

This study demonstrates that the COHIP-S is a reliable, valid, and culturally adapted tool for assessing OHRQoL in Sinhala-speaking adolescents with CLP living in Sri Lanka. Its excellent psychometric properties, including internal consistency and face, convergent, and discriminant validity, make it suitable for use in both TD adolescents and those with CLP. As a condition-specific instrument, it provides detailed insights into the impacts of orofacial anomalies, enabling SLTs and MDTs to monitor outcomes, identify QoL issues early, and make timely referrals. These findings represent an important step forward in evidence-based SLT practice in Sri Lanka, where validated QoL tools for children with CLP are limited.
